# Comparison between the Bouchama and Japanese Association for Acute Medicine Heatstroke Criteria with Regard to the Diagnosis and Prediction of Mortality of Heatstroke Patients: A Multicenter Observational Study

**DOI:** 10.3390/ijerph16183433

**Published:** 2019-09-16

**Authors:** Yutaka Kondo, Toru Hifumi, Junya Shimazaki, Yasutaka Oda, Shin-ichiro Shiraishi, Kei Hayashida, Tatsuma Fukuda, Masahiro Wakasugi, Jun Kanda, Takashi Moriya, Masaharu Yagi, Takashi Kawahara, Michihiko Tonouchi, Shoji Yokobori, Hiroyuki Yokota, Yasufumi Miyake, Keiki Shimizu

**Affiliations:** 1 Department of Emergency and Critical Care Medicine, Juntendo University Urayasu Hospital, Chiba 279-0021, Japan; 2 Department of Emergency and Critical Care Medicine, St. Luke’s International Hospital, Tokyo 104-8560, Japan; hifumitoru@gmail.com; 3 Department of Traumatology and Acute Critical Medicine, Osaka University Graduate School of Medicine, Osaka 565-0871, Japan; jshimazaki@nifty.com; 4 Advanced Medical Emergency and Critical Care Center, Yamaguchi University Hospital, Yamaguchi 755-8505, Japan; yasutaka-ygc@umin.ac.jp; 5 Department of Emergency and Critical Care Medicine, Aidu Chuo Hospital, Fukushima 965-8611, Japan; shinshi@nms.ac.jp; 6 Department of Emergency and Critical Care Medicine, School of Medicine, Keio University, Tokyo 160-8582, Japan; keilinda0714@gmail.com; 7 Department of Emergency and Critical Care Medicine, Graduate School of Medicine, University of the Ryukyus, Okinawa 903-0215, Japan; tatsumafukuda-jpn@umin.ac.jp; 8 Emergency and Critical Care Center, Toyama University Hospital, Toyama 930-0152, Japan; mwaka@med.u-toyama.ac.jp; 9 Department of Emergency Medicine, Teikyo University School of Medicine, Tokyo 173-8606, Japan; jkanda-cib@umin.ac.jp (J.K.); ymiyake-nsu@umin.ac.jp (Y.M.); 10 Department of Emergency and Critical Care Medicine, Saitama Medical Center, Jichi Medical University, Saitama 330-8503, Japan; tmoriya@jichi.ac.jp; 11 Department of Emergency and Critical Care Medicine, Urasoe General Hospital, Okinawa 901-2132, Japan; masaharuy0130@gmail.com; 12 Japan Sport Council, Tokyo 107-0061, Japan; kawahara2630@gmail.com; 13 Japan Meteorological Business Support Center, Tokyo 101-0054, Japan; tono@jmbsc.or.jp; 14 Department of Emergency and Critical Care Medicine, Nippon Medical School, Tokyo 113-8603, Japan; shoji@nms.ac.jp (S.Y.); yokota@nms.ac.jp (H.Y.); 15 Emergency and Critical Care Center, Tokyo Metropolitan Tama Medical Centre, Tokyo 183-8524, Japan; keishimizu-nsu@umin.ac.jp

**Keywords:** heat related illness, heat stroke, emergency, diagnosis

## Abstract

Background: This study aims to compare the Bouchama heatstroke (B-HS) and Japanese Association for Acute Medicine heatstroke (JAAM-HS) criteria with regard to the diagnosis and prediction of mortality and neurological status of heatstroke patients. Methods: This multicenter observational study recruited eligible patients from the emergency departments of 110 major hospitals in Japan from 1 July to 30 September, 2014. Results: A total of 317 patients (median age, 65 years; interquartile range, 39–80 years) were included and divided into the B-HS, JAAM-HS, and non-HS groups, with each group consisting of 97, 302, and 15 patients, respectively. The JAAM-HS (1.0; 95% confidence interval [CI], 0.87–1.0) and B-HS (0.29; 95% CI, 0.14–0.49) criteria showed high and low sensitivity to mortality, respectively. Similarly, the JAAM-HS (1.0; 95% CI, 0.93–1.0) and B-HS (0.35; 95% CI, 0.23–0.49) criteria showed high and low sensitivity to poor neurological status, respectively. Meanwhile, the sequential organ failure assessment (SOFA) scores demonstrated good accuracy in predicting mortality among heat-related illness (HRI) patients. However, both JAAM-HS and B-HS criteria could not predict in-hospital mortality. The AUC of the SOFA score for mortality was 0.83 (day 3) among the HRI patients. The patients’ neurological status was difficult to predict using the JAAM-HS and B-HS criteria. Concurrently, the total bilirubin level could relatively predict the central nervous system function at discharge. Conclusions: The JAAM-HS criteria showed high sensitivity to mortality and could include all HRI patients who died. The JAAM-HS criterion was considered a useful tool for judgement of admission at ED. Further investigations are necessary to determine the accuracy of both B-HS and JAAM-HS criteria in predicting mortality and neurological status at discharge.

## 1. Background

Heatstroke (HS) is one of the most serious and life-threatening conditions that are directly related to heat exposure [1,2,3]. HS patients typically present with an increased core body temperature (BT) and central nervous system dysfunction, which can often become fatal in the case of inadequate or late treatment [4,5]. Moreover, even if the patient survives, he or she may still sustain severe neurological disorders and even become bedridden. Therefore, the diagnosis and treatment of HS patients is important.

**Figure 1:**
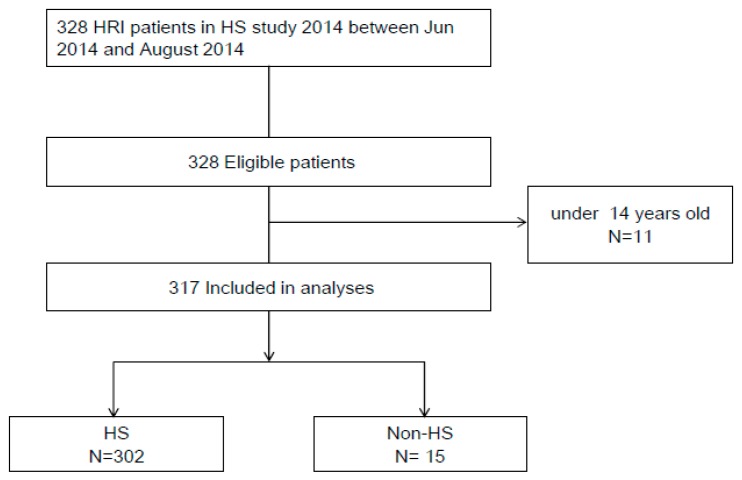
Patient enrollment and exclusion process of the study.

In 2002, Bouchama et al. are developed a set of criteria to accurately diagnose HS and explore potential therapeutic and preventive strategies for this condition [4]. The Bouchama heatstroke (B-HS) criteria clinically define HS as a core BT that increases to >40°C and is accompanied by hot, dry skin, and central nervous system abnormalities such as delirium, convulsions, or coma [4]. Additionally, the B-HS criteria specify that HS should result from exposure to a high environmental temperature (called classic or non-exertional HS) or strenuous exercise (called exertional HS) [4] (Figure 1). This definition is clear and easy to use and has been utilized in clinical settings for a long time.

However, a number of HS clinical criteria indicate that this condition also results in the occurrence of multiple organ failure (MOF), leading to death, and is treated using several kinds of cooling treatment and other management approaches [6,7,8,9,10,11,12]. However, the B-HS criteria only include the development of central nervous system disorders due to HS, and death cases may not be attributed to HS because other organ damage markers are not included in the criteria. To address this concern, the Japanese Association for Acute Medicine (JAAM) developed a new HS criteria that include components that cover the presence of renal and hepatic complications and disseminated intravascular coagulation (DIC) (Figure 1) [13,14]. Additionally, the Japanese Association for Acute Medicine heatstroke (JAAM-HS) criteria do not include temperature because elderly people often do not present with high temperature even when they are already suffering from HS.

So far, studies on the comparison between B-HS and JAAM-HS criteria remain to be conducted. Thus, this study aims to clarify the differences between the B-HS and JAAM-HS in terms of the diagnosis and identification of mortality and neurological outcomes among HS patients.

## 2. Methods

### 2.1. Study Design and Patients’ Enrolment

This multicenter observational study was performed and analyzed. In brief, 110 major hospitals in Japan were prospectively registered in this study, and eligible patients were recruited from the emergency departments of these hospitals from 1 July to 30 September, 2014. Patients who had heat-related illness (HRI) based on the judgment of a medical doctor and were admitted to a hospital for HRI treatment were enrolled in this study. We excluded those patients who do not need to be admitted for HRI treatment. The data were manually recorded by a staff member or medical doctor at each participating hospital using specific record sheets. The study protocol was approved by the Ethics Committees of Showa University Hospital. This study was conducted in accordance with the ethical standards established in the 1964 Declaration of Helsinki and its later amendments. The requirement for patient consent was waived due to the observational nature of the study.

### 2.2. Definitions 

We used both the B-HS and JAAM-HS criteria for the diagnosis of HS in this study (Table 1) [15,16,17,18]. JAAM Heat Stroke Committee (JAAM-HS) launched a working group (JAAM-HS-WG) to analyze the collected data regarding heat-related illness in 2016. JAAM-HS-WG simplified the heat stroke classification (Table 1). Furthermore, the clinician checked for HS by allowing the patient to cool down first before the body temperature (BT) was measured. DIC was diagnosed based on the Japanese Association for Acute Medicine DIC scoring system, where DIC is diagnosed if the total score is ≥4 [15,16,17,18]. In brief, this scoring system consists of the following 4 components: systemic inflammatory response syndrome, platelet count, prothrombin time (PT), and fibrin/fibrinogen degradation product (FDP) or d-dimer level (Additional file 1). The missing data were scored as zero.

Meanwhile, we used the modified Rankin Scale (mRS) to evaluate the neurological function of the patients [19,20,21,22]. The mRS consists of 7-level ordered categorical scales rated as follows: 0, no symptoms at all; 1, no significant disability—possible to perform all usual duties and activities despite symptoms; 2, slight disability—impossible to perform all previous activities, but possible to look after own affairs without assistance; 3, moderate disability—possible to walk without assistance, but requiring some help; 4, moderately severe disability—unable to walk without assistance and unable to attend by own without assistance; 5, severe disability—bedridden, incontinent, and requiring constant nursing care and attention; and 6, dead [23]. We also defined that poor neurological status is mRS pointed 3 to 6.

### 2.3. Data Sampling

Data on the following variables were collected: age, sex, presence of preexisting diseases (cardiac, psychiatric, and cerebrovascular disorders; hypertension; and diabetes mellitus), vital signs on arrival and during prehospital period, laboratory values on arrival (arterial blood gas, white blood cell count, hemoglobin, hematocrit, platelet count, blood urea nitrogen [BUN], creatinine [Cre], total bilirubin [T.Bil], aspartate transaminase, alanine transaminase, creatinine kinase, sodium, potassium, chloride, glucose, PT ratio, FDP, and antithrombin III), and hospital mortality. Especially the superficial BT (axilla and ear) was measured on the scene, whereas both the superficial and core BTs (rectum) were measured at the emergency department (ED).

### 2.4. Statistical Analysis

We excluded patients who were under 14 years old with HS from analysis. The continuous measurement variables were expressed as the median and interquartile range (IQR, 25–75%). The JMP Pro software, version 13 (SAS Institute, Cary, NC, USA) was used for the statistical analyses and calculations. Comparisons between the two groups were made using the unpaired student’s *t*-test and Mann–Whitney’s U-test for parametric and nonparametric data, respectively, and either the Pearson’s chi-square or Fisher’s exact test was used when required. We depicted the receiver operating characteristic (ROC) curves and calculated the area under the curve (AUC) to evaluate the accuracy of the sequential organ failure assessment (SOFA) score, T.Bil level, and B-HS and JAAM-HS criteria in predicting the mortality and neurological status of HRI patients. Differences with *p* < 0.05 were considered statistically significant.

## 3. Results

In total, 328 patients were consecutively enrolled in this study. Eleven patients were excluded because they were aged >14 years. The remaining 317 patients were classified either as HS or non-HS patients (Figure 1). The HS patients were further categorized into the B-HS or JAAM-HS group based on the HS criteria (Table 1). 

### 3.1. Baseline Characteristics of The Study Population

A total of 317 patients (median age, 65 years; IQR, 39–80 years) were included in this study, with a male-to-female ratio of 229:88. The B-HS, JAAM-HS, and non-HS groups comprised 97, 302, and 15 HRI patients, respectively. Table 2 shows a comparison of the clinical conditions between the B-HS, JAAM-HS, and non-HS patients. In brief, the JAAM-HS and non-HS groups were statistically significant difference in terms of age; heart rate; Glasgow coma scale (GCS) score; BUN, Cre, and Na levels; and SOFA score. 

### 3.2. Clinical Manifestations of B-HS, JAAM-HS, and Non-HS Patients

Sweat was the most common symptom (121 patients, 38.2%) observed among the participants in this study. This symptom was observed among the 31 (32%), 113 (37.4%), and 8 (53.3%) patients from the B-HS, JAAM-HS, and non-HS groups, respectively (Table 3). Meanwhile, general fatigue was the second most relevant symptom (103 patients, 32.5%), with 22 (22.7%), 95 (31.5%), and 8 (53.3%) patients from the B-HS, JAAM-HS, and non-HS groups, respectively, manifesting this symptom. In contrast, the gastrointestinal (GI) symptoms are the least common type of symptom observed in this population. Only 8 (2.5%), 7 (2.2%), and 9 (2.8%) patients from the B-HS, JAAM-HS, and non-HS groups, respectively, had diarrhea, abdominal pain, and incontinence. The presence of musculoskeletal symptoms were determined based on whether the patients experienced muscle pain and cramp. A total of 6 (6.2%), 40 (13.2%), and 2 (13.3%) patients from the B-HS, JAAM-HS, and non-HS groups, respectively, had muscle pain. Meanwhile, 10 (9.7%), 64 (21.2%), and 2 (13.3%) patients from the B-HS, JAAM-HS, and non-HS groups, respectively, experience muscle cramp. Headache was commonly observed in the non-HS group (5 patients, 33.3%; *p* < 0.001). In contrast, only 5 (5.5%) and 19 (6.3%) patients from the B-HS and JAAM-HS groups, respectively, had headache.

### 3.3. Prediction of Mortality among HRI Patients

A total of 6 (6.2%) and 21 patients (7.0) from the B-HS and JAAM-HS groups, respectively, died (no significance [NS], *p* = 0.79). Meanwhile, none of the patients from the non-HS group died. The sensitivity to death of the evaluated criteria was 1.0 (95% CI, 0.87–1.0) in the JAAM-HS group, whereas it was 0.29 (95% CI, 0.14–0.49) B-HS group. Meanwhile, the specificity to death of the evaluated criteria was 0.69 (95% CI, 0.68–0.71) and 0.05 (95% CI; 0.04–0.05) in the B-HS and JAAM-HS groups, respectively (Table 4). The positive likelihood ratio (LR+) of death was 0.93 (95% CI, 0.44–1.68) and 1.05 (95% CI, 0.91–1.05) in the B-HS and JAAM-HS groups, respectively. Furthermore, the negative likelihood ratio (LR−) of death was 1.03 (95% CI, 0.72–1.26) and 0 (95% CI, 0–3.19) in the B-HS and JAAM-HS groups, respectively. The Youden index [24,25] was −0.22 (95% CI, −0.18 to 0.2) and 0.05 (95% CI, −0.09 to 0.05) in the B-HS and JAAM-HS groups, respectively. In contrast, the AUC values were 0.52 in both B-HS and JAAM-HS groups, respectively (NS, *p* = 0.97). The odds ratio (OR) was 0.90 (95% CI, 0.35–2.33) in the B-HS group. The OR in the JAAM-HS group could not be calculated. 

**Figure 2:**
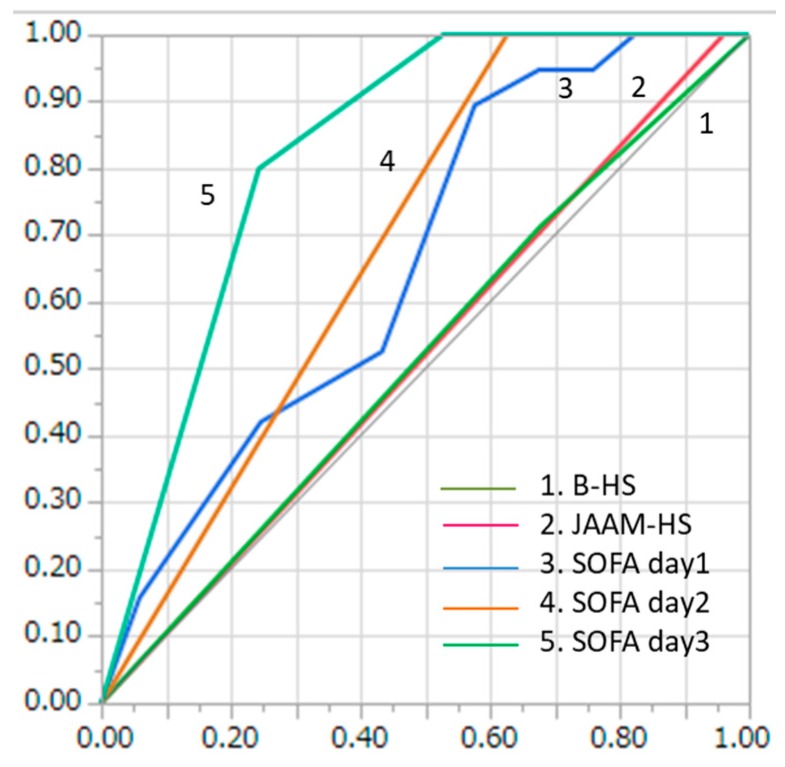
Comparison of AUC for in-hospital mortality between the B-HS and JAAM-HS patients.

The consecutive SOFA scores measured showed good accuracy in predicting mortality among HRI patients. The AUC values of the SOFA scores for mortality were 0.66 (on admission), 0.69 (day 2), and 0.83 (day 3) among the HRI patients (Figure 2). With regard to the prediction of mortality, SOFA score (day 3) showed significantly better accuracy than the B-HS criteria (*p* < 0.01). However, no statistically significant differences in mortality predictive accuracy were observed between the B-HS criteria and SOFA scores on admission and day 2 (on admission, *p* = 0.11; day 2, *p* = 0.13). Meanwhile, all in-hospital SOFA scores demonstrated better accuracy than the JAAM-HS criteria in predicting the mortality of the HRI patients (on admission, *p* = 0.02; day 2, *p* = 0.04; day 3, *p* < 0.001).

### 3.4. Prediction of Neurological Function among HRI Patients

A total of 14 (6.2%) and 40 (13.2%) patients from the B-HS and JAAM-HS groups, respectively, had poor neurological status (PNS) (NS, *p* = 0.76). None of the patients from the non-HS group had PNS. The sensitivity to PNS of the evaluated criteria was 1.0 (95% CI, 0.93–1.0) JAAM-HS group, whereas it was 0.35 (95% CI, 0.23–0.49) B-HS group. Meanwhile, the specificity to PNS of the evaluated criteria was 0.70 (95% CI, 0.68–0.72) and 0.05 (95% CI, 0.04–0.05) in the B-HS and JAAM-HS groups, respectively (Table 5). Moreover, the LR+ of PNS was 1.17 (95% CI, 0.72–1.77) and 1.06 (95% CI, 0.97–1.06) in the B-HS and JAAM-HS groups, respectively. In contrast, the LR− of PNS was 0.93 (95% CI, 0.70–1.13) and 0 (95% CI, 0–1.66) in the B-HS and JAAM-HS groups, respectively. The Youden index (18, 19) was 0.05 (95% CI, −0.09 to 0.21) and 0.05 (95% CI, −0.03 to 0.05) in the B-HS and JAAM-HS groups, respectively. Furthermore, the AUC values were 0.52 and 0.53 in the B-HS and JAAM-HS groups, respectively (NS, *p* = 0.95). Concurrently, the OR was 1.26 (95% CI, 0.63–2.51) in the B-HS group. However, this value could not be determined in the JAAM-HS group. 

**Figure 3:**
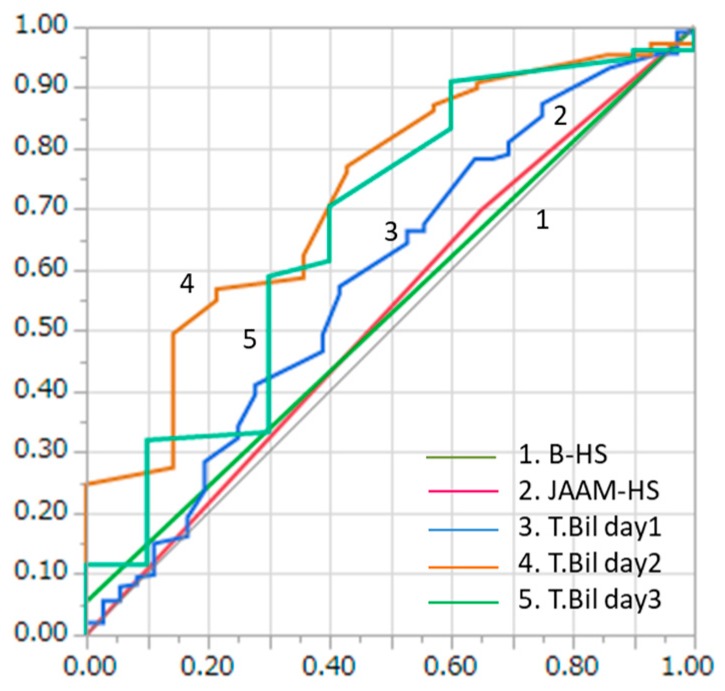
Comparison of AUC for neurological outcomes between the B-HS and JAAM-HS patients.

The consecutive serum T.Bil levels measured showed good accuracy in predicting the PNS of HRI patients. The AUC values of the serum T.Bil levels for PNS were 0.58 (on admission), 0.72 (day 2), and 0.67 (day 3) among the HRI patients (Figure 3). The serum T.Bil levels (day 2) demonstrated significantly better accuracy in predicting PNS than the B-HS criteria (*p* < 0.01). On the contrary, no statistically significant differences in PNS predictive accuracy were observed between the B-HS criteria and serum T.Bil levels on admission and day 3 (on admission, *p* = 0.32; day 2, *p* = 0.17). Meanwhile, the serum T.Bil levels on day 2 had better PNS predictive accuracy among the HRI patients than the JAAM-HS criteria (*p* < 0.01). In contrast, no statistically significant differences in PNS predictive accuracy were found between the JAAM-HS criteria and serum T.Bil levels on admission and day 3 (on admission, *p* = 0.31; day 3; *p* = 0.17).

## 4. Discussion

HS is a serious clinicopathophysiologic condition that can lead to MOF, poor neurological outcomes, and even death [26]. In this study, we first evaluated the JAAM-HS criteria by comparing it with the B-HS criteria among the for HRI patients. The JAAM-HS criteria included hepatic and renal dysfunction and DIC score components, which indicates that we can only use this criteria the hospital settings (Table 1). In contrast, the B-HS criteria can be used in prehospital settings because it only requires physical examination. 

The characteristics of each patient group (B-HS, JAAM-HS, and non-HS) are well classified based on the different criteria in this study. The median SOFA scores were 5, 3, and 0 in the B-HS, JAAM-HS, and non-HS, groups, respectively (Table 2), which implies that the criteria were ordered based on the severity of HRI. In Table 3, sweat was the most common symptom (121 patients, 38.2%) observed among all patients, and this symptom was found in 31 (32%), 113 (37.4%), and 8 (53.3%) patients from the B-HS, JAAM-HS, and non-HS groups, respectively (Table 3). Meanwhile, only 8 (2.5%), 7 (2.2%), and 9 (2.8%) patients from the B-HS, JAAM-HS, and non-HS groups, respectively, experienced GI symptoms, such as diarrhea, abdominal pain, and incontinence (Table 3). HS is known to possibly increase the occurrence of GI symptoms, although the mechanism behind this finding is not fully understood. Some researchers insist that heat and exercise may cause immunosuppression in the gut [27,28], whereas others claim that these GI symptoms may be simply caused by drinking too much water. In either case, we did not observe these GI symptoms among the participants in this study. We collected only the data of the HRI patients who were admitted, and the occurrence of these GI symptoms may be increased among outpatients. These GI symptoms appear to be relatively mild clinical manifestation of HS, which need further investigation. In total, the B-HS group showed less symptoms in most cases, except for agitation, diarrhea, abdominal pain, and incontinence, than the JAAM-HS and non-HS groups. These results may indicate that the B-HS patients are suspected to be unable to complain their symptoms because they have reduced consciousness. Actually, the median GCS scores of B-HS, JAAM-HS, and non-HS patients were 10, 14, and 15, respectively (Table 2). 

Prompt cooling is the cornerstone of HS treatment, and the prognosis of HS patients relies on this treatment if the BT is 40.0 °C (104 °F) or more [26,29,30]. Despite the absence of strong evidences, some studies demonstrated that the mortality rate may decrease if the BT is reduced to <40.0 °C (104 °F) within 30 min after the loss of consciousness [26,29]. Bouchama et al. (2002) included BT in their B-HS criteria, and increased focus is given in the immediate diagnosis and cooling of this population [4]. However, in this study, we still observed a high mortality rate even in patients of the JAAM-HS group who had <40 °C BT JAAM-HS group. Specifically, the mortality rates were 7.0% and 6.2% in the JAAM-HS and B-HS groups, respectively (Table 4). Additionally, no significant differences in the neurological outcomes were found between the B-HS (14.4%) and JAAM-HS (13.2%) groups. In fact, the BTs of the JAAM-HS group were 38.3 °C on the scene and 38.8 °C in the ED of the admitted. On the contrary, the B-HS group displayed considerably higher BT than the JAAM-HS group, which were 40.1 °C both on the scene and in the ED of the admitted hospitals (Table 2). These findings imply that high temperature may be associated with poor prognosis [31], although no obvious evidence exists suggesting 40 °C as the cutoff point for BT. Hence, we may need to reconsider changing the cutoff value from 40 °C to approximately 38.8 °C to include patients from the JAAM-HS group who died. 

One of the impressive results of this study was that the JAAM-HS criteria included all HS patients who died, which is clinically meaningful. The use of the JAAM-HS criteria in the ED can be a milestone for patients who need admission or are going home. Meanwhile, the B-HS criteria only included 6 (29%) patients who died, which suggests that it may not be accurate for the diagnosis of HS. Almost all patients who died should be diagnosed with HS because this condition is associated with multiorgan dysfunction caused by heat [32,33]. 

We found that both the JAAM-HS and B-HS criteria could not predict in-hospital mortality (AUC: 0.52 for both the JAAM-HS and B-HS criteria) because there were too many latent factors that make it difficult to predict mortality from heat stress. This result suggested that these criteria were only useful for the diagnosis of HS. Meanwhile, the SOFA scores predicted the mortality (AUC: 0.83 for SOFA day 3) of the HRI patients well. This result is consistent with the findings of other studies showing that SOFA scores can predict mortality well among critically ill patients in the ICU setting [34,35,36]. Additionally, the SOFA scores showed increased predictive accuracy as the hospital stay lengthened (AUC: 0.66, 0.69, and 0.83 for SOFA score days 1, 2, and 3, respectively) because the severity of HS became clearer over time. We also evaluated the neurological functions of the HRI patients at discharge using the mRS. The neurological status was difficult to predict through the JAAM-HS and B-HS criteria (AUC: 0.53 and 0.52 for the JAAM-HS and B-HS criteria, respectively). Concurrently, the T.Bil level could relatively predict the central nervous system function at discharge (AUC: 0.72 for T.Bil level day 2) compared with the JAAM-HS and B-HS criteria. However, even the T.Bil level showed insufficient accuracy, and we hope that a new marker that can predict the neurological status of HS patients will emerge. 

**Figure 4:**
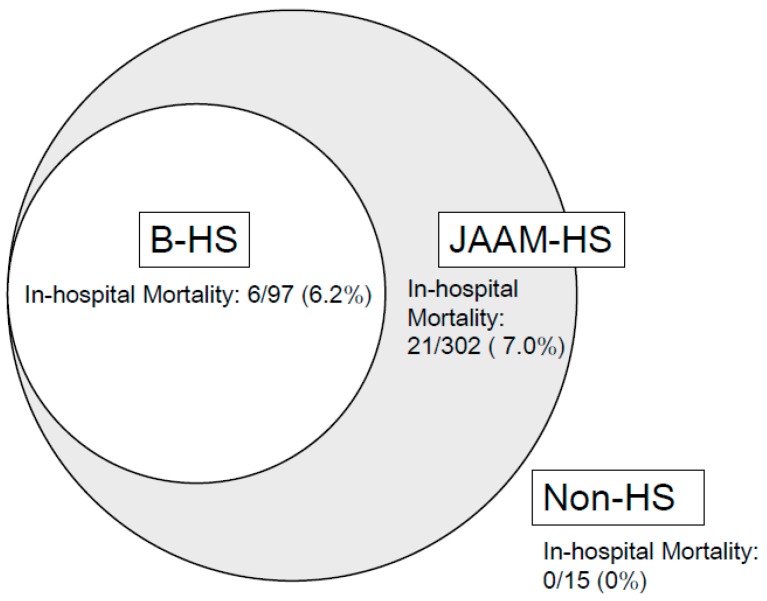
Distribution of HS population based on the B-HS and JAAM-HS criteria.

Figure 4 presents the distribution of the patients into either the B-HS, JAAM-HS, or non-HS group. The components of the B-HS criteria are completely covered by the JAAM-HS criteria. Additionally, the results of this study revealed that none of the patients from the non-HS group died. The B-HS criteria were unable to include all patients with fatal HS, but the JAAM-HS criteria may include even the non-HS population. We hope that new diagnostic HS criteria that can be used as a mortality prediction tool will be developed and the borderline between the B-HS and JAAM-HS criteria will become clear.

This study has several limitations to consider when interpreting the results. First, the presence of exertional or non-exertional HS could not be distinguished in this study, which may lead to biases in the results. Second, the number of patients who died was only 21 because we performed this study only from July to September. Additionally, the winter and spring seasons in Japan indicate the decreased morbidity associated with HS. We could not get enough data on the outcomes, and hence, this study may need to be continued for a longer period of time. Third, we excluded child population because of the aim of the study. It may become different results if we include child population. The persons less than 14-years-old are the most vulnerable sector of population due to the immaturity of their thermoregulatory mechanisms. Fourth, this is an observational study, which indicates that the treatments for HRI both in the ED and after admission could not be controlled. Especially, body cooling is one of the considerable treatments for HRI, which can contribute to mortality and development of neurological conditions; that is, the delay of the cooling treatment can affect the outcomes. Finally, this study was performed only in Japanese hospitals. Hence, it needs external validation because each country has different atmospheric temperatures [37], which implies that the prevalence of disease and mortality can vary.

## 5. Conclusions

We compared the B-HS and JAAM-HS criteria with regard to the diagnosis and prediction of mortality and neurological status of HS patients. Both B-HS and JAAM-HS were unable to predict mortality and PNS at discharge. The JAAM-HS criteria showed high sensitivity to mortality and is considered to be a useful tool for the judgement of admission for HRI patients at ED.

## Figures and Tables

**Table 1:** Diagnostic criteria for the B-HS and JAAM-HS groups.

Criteria	Definition
B-HS	It is diagnosed by meeting all three below criteria
1. Core temperature > 40 °C	
2. Central nervous system abnormalities (such as delirium, convulsions, or coma) or documented evidence of cooling before the first record temperature	
3. A reliable history of compatible environmental exposure (hot, dry or flushed skin)	
JAAM-HS	Physician suspect heat disorder and show one or more below criteria
1. Central nervous system abnormalities (GCS ≤ 14)	
2. Hepatic/Renal dysfunction (T.Bil ≥ 1.2, Cre ≥ 1.2)	
3. Coagulation disorder (DIC, JAAM criteria *)	

B-HS, Bouchama heatstroke; JAAM-HS, Japanese Association for Acute Medicine heatstroke; GCS, Glasgow coma score; T.Bil, total bilirubin; Cre, creatinine; DIC, disseminated intravascular coagulation. * Supplement File S1.

**Table 2:** Characteristics of the B-HS, JAAM-HS, and non-HS patients.

Variables	B-HS	JAAM-HS	Non-HS	*p*-Value
Age	71 (52–82)	66 (41–81)	20 (15–53)	<0.001 ***
Gender (male/female)	66/31	217/85	12/3	0.76
sBP (mmHg)	121 (102.8–145.3)	123 (107–143)	138 (127–147)	0.20
dBP (mmHg)	70 (57–81)	71 (62–87)	75 (70–85)	0.26
HR (bpm)	114 (82.5–141.5)	98 (77–120)	78 (68–103)	0.04 *
RR (/min)	24 (20–35)	21 (17–30)	18 (15–24)	0.12
SpO_2_ (%)	98 (96–99)	98 (96–99)	98 (97–99)	0.4
BT (°C, On-scene)	40.1 (38.2–41)	38.3 (36.7–40)	37.0 (36.6–39.7)	0.17
BT (°C, ER, superficial)	39.1 (37.3–40.3)	37.1 (36.4–38.7)	36.8 (36.4–37.6)	0.28
BT (°C, ER, core body)	40.1 (38.8–41)	38.8 (37.4–40.1)	38.8 (37.2–40)	0.82
GCS	10 (3–15)	14 (10–15)	15	<0.001 ***
pH	7.43 (7.38–7.48)	7.42 (7.38–7.46)	7.46 (7.42–7.48)	0.35
PaO_2_ (mmHg)	109.7 (72–186.9)	94.8 (67.1–148.5)	65.2 (32–94.5)	0.28
PaCO_2_ (mmHg)	32.2 (25.8–39.1)	35.3 (32.5–39.9)	35.3 (32.5–39.9)	0.99
WBC (/μL)	10,200 (7400–13,420)	10,415 (7882.5–14,000)	9450 (6260–10,185)	0.08
Hgb (g/dl)	13.6 (11.8–15.4)	14.3 (12.5–16.3)	14.8 (13.2–15.6)	0.76
Hct (%)	40.4 (35.3–45.9)	42.5 (37.1–47.3)	42.8 (37.7–44.2)	0.19
Plt (×10^3^/μL)	20.1 (15.8–24.9)	22.1 (4.6–50.8)	20.3 (19.7–35.7)	0.75
BUN (mg/dl)	23.2 (17.6–34.6)	24.5 (18–34)	14.4 (10.1–19.2)	0.04 *
Cre (mg/dl)	1.41 (1.04–2.02)	1.51 (1.03–2.29)	0.81 (0.65–0.97)	<0.01 **
T.Bil (mg/dl)	0.9 (0.7–1.4)	0.9 (0.7–1.3)	0.8 (0.7–0.9)	0.24
AST (U/L)	46 (28–107)	33 (26–62.5)	27 (21–40)	0.33
ALT (U/L)	30 (18–70)	25 (17–51)	15 (13–35)	0.45
CK (U/L)	337 (142–853)	290.5 (133.5–621.3)	166 (107.5–259)	0.46
Na (mmol/L)	140 (135–144)	140 (136–143)	138.5 (134–140.8)	0.04 *
K (mmol/L)	4.1 (3.7–4.7)	4.1 (3.7–4.7)	3.8 (3.6–4.3)	0.20
Cl (mmol/L)	103 (99–108)	102 (99–106)	103 (98–107)	0.33
Glucose (mg/dl)	160 (124–205)	145 (51–183)	115 (100–136)	0.86
PT ratio	1.1 (1.03–1.24)	1.08 (1–1.17)	1.05 (1.03–1.11)	0.36
FDP (μg/mL)	9.3 (3.3–46.7)	5.15 (2.7–13.8)	3 (3–3.75)	0.35
D-dimer (μg/mL)	5 (1.2–17.9)	2.2 (0.8–7.65)	0.6 (0.5–4.7)	0.35
AT Ⅲ (%)	97.5 (80.8–109)	101 (86.9–111.4)	95.5 (84.3–142)	0.5
DIC score	1 (0–4)	0 (0–1)	0 (0–1)	0.14
SOFA score	5 (2–9)	3 (2–5)	0 (0–1)	<0.001 ***
	median, (interquartile range)			JAAM-HS vs. non-HS

B-HS, Bouchama heatstroke; JAAM-HS, Japanese Association for Acute Medicine heatstroke; HS, heatstroke; BP, blood pressure; HR, heart rate; RR, respiratory rate; BT, body temperature; WBC, white blood cells; GCS, Glasgow coma score; Cre, creatinine; T.Bil, total bilirubin; PT, prothrombin time; FDP, fibrin/fibrinogen degradation product; AT III, antithrombin III; DIC, disseminated intravascular coagulation; SOFA, sequential organ failure assessment. * *p* < 0.05, ** *p* < 0.01, *** *p* < 0.001.

**Table 3:** Clinical manifestations of the B-HS, JAAM-HS, and non-HS patients.

Symptom	B-HS	JAAM-HS	Non-HS	Total	*p*-Value
thirsty	8 (8.2)	36 (11.9)	2 (13.3)	38 (12)	0.86
muscle pain	6 (6.2)	40 (13.2)	3 (20)	43 (13.6)	0.56
muscle cramp	10 (9.7)	64 (21.2)	2 (13.3)	66 (20.8)	0.46
dizziness	6 (6.2)	27 (8.9)	1 (6.7)	28 (8.8)	0.76
general fatigue	22 (22.7)	95 (31.5)	8 (53.3)	103 (32.5)	0.08
syncope	0 (0)	11 (3.6)	1 (6.7)	12 (3.8)	0.55
headache	5 (5.2)	19 (6.3)	5 (33.3)	24 (7.6)	<0.001 ***
agitation	6 (6.2)	14 (4.6)	1 (6.7)	15 (4.7)	0.72
sweat	31 (32)	113 (37.4)	8 (53.3)	121 (38.2)	0.37
vomit	14 (14.4)	52 (17.2)	3 (20)	55 (17.4)	0.78
diarrhea	2 (2.1)	8 (2.6)	0 (0)	8 (2.5)	0.52
abdominal pain	2 (2.1)	6 (2)	1 (6.7)	7 (2.2)	0.23
incontinence	5 (5.2)	9 (3)	0 (0)	9 (2.8)	0.5
	N, (%)				

B-HS, Bouchama heatstroke; JAAM-HS, Japanese Association for Acute Medicine heatstroke; HS, heatstroke. JAAM-HS vs. non-HS, *** *p* < 0.001.

**Table 4:** Prediction of mortality among the B-HS and JAAM-HS patients.

	B-HS	JAAM-HS
Mortality, Number (%)	6/97 (6.2)	21/302 (7.0)
Sensitivity of death (95% CI)	0.29 (0.14–0.49)	1.0 (0.87–1.0)
Specificity of death (95% CI)	0.69 (0.68–0.71)	0.05 (0.04–0.05)
LR+	0.93 (0.44–1.68)	1.05 (0.91–1.05)
LR−	1.03 (0.72–1.26)	0 (0–3.19)
Youden index	−0.22 (−0.18–0.2)	0.05 (−0.09–0.05)
AUC	0.52	0.52
OR (95% CI)	0.90 (0.35–2.33)	Inf

B-HS, Bouchama heatstroke; JAAM-HS, Japanese Association for Acute Medicine heatstroke; CI, confidence interval; LR, likelihood ratio; AUC, area under the curve; OR, odds ratio.

**Table 5:** Prediction of neurological outcomes between the B-HS and JAAM-HS patients.

	B-HS	JAAM-HS
PNS ([mRS, 3–6]), Number (%)	14/97 (14.4)	40/302 (13.2)
Sensitivity of PNS (95% CI)	0.35 (0.23–0.49)	1.0 (0.93–1.0)
Specificity of PNS (95% CI)	0.70 (0.68–0.72)	0.05 (0.04–0.05)
LR+	1.17 (0.72–1.77)	1.06 (0.97–1.06)
LR−	0.93 (0.70–1.13)	0 (0–1.66)
Youden index	0.05 (−0.09–0.21)	0.05 (−0.03–0.05)
AUC	0.52	0.53
OR (95% CI)	1.26 (0.63–2.51)	Inf

B-HS, Bouchama heatstroke; JAAM-HS, Japanese Association for Acute Medicine heatstroke; PNS, poor neurological status; mRS, modified Rankin scale; CI, confidence interval; LR, likelihood ratio; AUC, area under the curve; OR, odds ratio.

## Supplementary Materials

The following are available online at https://www.mdpi.com/1660-4601/16/18/3433/s1, File S1: the Japanese Association for Acute Medicine criteria for the diagnosis of disseminated intravascular coagulation.

## Abbreviations

AUC	area under the curve
B-HS	Bouchama heatstroke
BT	body temperature
BUN	blood urea nitrogen
CI	confidence interval
Cre	creatinine
DIC	disseminated intravascular coagulation
ED	emergency department
FDP	fibrin/fibrinogen degradation product
GCS	Glasgow coma score
HRI	heat-related illness
HS	heatstroke
JAAM-HS	Japanese Association for Acute Medicine heatstroke
MOF	multiple organ failure
mRS	modified Rankin scale
PNS	poor neurological status
PT	prothrombin time
ROC	receiver operating characteristic
SOFA	sequential organ
T.Bil	total bilirubin
