# Supplementary material for: Comparison between the Bouchama and Japanese Association for Acute Medicine Heatstroke Criteria with Regard to the Diagnosis and Prediction of Mortality of Heatstroke Patients: A Multicenter Observational Study
Source: Int J Environ Res Public Health. 2019 Sep 16;16(18):3433. doi: 10.3390/ijerph16183433 (PMC6765926; doi:10.3390/ijerph16183433)
Supplement: Supplementary file 1 [file ijerph-16-03433-s001.pdf]

Supple 1.

Criteria for DIC	Score
Systemic inflammatory response syndrome criteria*	
≧ 3	1
0-2	0
Platelet counts (10^9/L)	
< 80 or more than 50% decrease within 24 hours	3
≧ 80, <120 or more than 30% decrease within 24 hours	1
≧ 120	0
Prothrombin time (value of patient/normal value)	
≧ 1.2	1
< 1.2	0
Fibrin/fibrinogen degradation products (mg/L)	
≧ 25	3
≧ 10, <25	1
< 10	0
Diagnosis of DIC	
4 points or more	

*Criteria for *systemic inflammatory response syndrome*

1. Temperature >38 °C or <36 °C
2. Heart rate >90 beats/min
3. Respiratory rate >20 breath /min or $PaCO_2$≦32 torr
4. White cell blood counts >12,000/mm^3, <4,000cells/mm^3, or 10% immature (band) forms
